# Update zur Regressionsgraduierung nichtkleinzelliger Lungenkarzinome

**DOI:** 10.1007/s00292-026-01544-z

**Published:** 2026-02-24

**Authors:** Felix Elsner, Lena-Maria Löwer-Kiem, Florian Fuchs, Sabina Berezowska, Konrad Steinestel

**Affiliations:** 1 Institut für Pathologie, Universitätsklinikum Erlangen, Friedrich-Alexander-Universität Erlangen-Nürnberg, Erlangen, Deutschland; 2 Institut für Pathologie und Molekularpathologie, Bundeswehrkrankenhaus Ulm, Ulm, Deutschland; 3 Medizinische Klinik 1, Universitätsklinikum Erlangen, Friedrich-Alexander-Universität Erlangen-Nürnberg, Erlangen, Deutschland; 4 Institut Universitaire de Pathologie, Centre Hospitalier Universitaire Vaudois et Université de Lausanne, Lausanne, Schweiz

**Keywords:** Lungenkrebs, Therapieansprechen, Checkpoint-Inhibition, Tumorregression, Neoadjuvante Chemoimmuntherapie, Lung cancer therapy response, Checkpoint inhibition, Tumor regression, Neoadjuvant chemoimmunotherapy

## Abstract

Immuncheckpoint-Inhibitoren in Kombination mit konventioneller Chemotherapie haben im neoadjuvanten und perioperativen Setting des nichtkleinzelligen Lungenkarzinoms (NSCLC) zu deutlich verbesserten onkologischen Ergebnissen geführt und sind als Standardtherapie für Patienten in den UICC-Stadien IIA–IIIA etabliert. Damit gewinnt die pathologische Beurteilung der therapieinduzierten Tumorregression an Bedeutung. Der Artikel gibt einen Überblick über die aktuellen Empfehlungen zur makroskopischen Aufarbeitung, histologischen Bewertung und zum Staging von NSCLC-Resektaten nach neoadjuvanter Chemoimmuntherapie. Wesentliche Aspekte umfassen die makroskopische Aufarbeitung des Tumorbettes, die quantitative Erfassung der Tumorbettkomponenten (vitaler Tumor, Stroma/Entzündung, Nekrose) in 10 %-Schritten sowie die Anwendung etablierter Regressionsgraduierungen (IASLC, Junker). Zudem werden Herausforderungen bei der Abgrenzung therapieinduzierter Veränderungen, der Bewertung von Lymphknoten und der Stadieneinteilung nach neoadjuvanter Therapie erläutert. Die aktuelle Datenlage unterstreicht die Notwendigkeit einer standardisierten Vorgehensweise bei der pathologischen Diagnostik sowie weiterer Untersuchungen, um prädiktive Biomarker zu identifizieren und die prognostische Aussagekraft der pathologischen Regressionsgraduierung zu verbessern.

## Abstract (en)

Immune checkpoint inhibitors combined with conventional chemotherapy have led to significantly improved outcomes in the neoadjuvant and perioperative treatment of non-small cell lung cancer (NSCLC), establishing chemoimmunotherapy as the standard of care for patients in UICC stages IIA–IIIA. As a result, pathological assessment of therapy-induced tumor response has become essential. This article provides an overview of the current recommendations for macroscopic handling, histological evaluation, and staging of NSCLC resection specimens after neoadjuvant treatment. Key aspects include comprehensive embedding of the tumor bed, quantitative assessment of tumor bed components (viable tumor, stroma/inflammation, necrosis) in 10% increments, and the application of established regression grading systems (IASLC, Junker). Challenges in distinguishing therapy-induced changes, evaluating lymph nodes, and determining post-treatment staging are discussed. Current findings highlight the need for standardized diagnostic procedures and further research to identify predictive biomarkers and improve the prognostic value of pathological regression grading after neoadjuvant chemoimmunotherapy.

Immuncheckpoint-Inhibitoren in Form monoklonaler Antikörper gegen PD‑1 („programmmed cell death protein 1“) oder PD-L1 („programmmed cell death ligand 1“) führen zu einer Verstärkung bzw. Wiederherstellung der gegen die Krebszellen gerichteten T‑Zell-Antwort [32]. Nach deutlichen Therapieerfolgen in fortgeschrittenen und metastasierten Stadien des nicht-kleinzelligen Lungenkarzinoms (NSCLC), haben mehrere Studien im neoadjuvanten oder perioperativen Setting [4, 12, 13, 20, 28] ein signifikant verbessertes ereignisfreies Überleben und Gesamtüberleben für die Kombination aus Immuntherapie und konventioneller Chemotherapie gezeigt, was dazu führte, dass sich die neoadjuvante Chemoimmuntherapie zur Standardtherapie für operable NSCLC-Patienten ab dem UICC-Stadium IIA entwickelt hat [31]. Insbesondere war die Rate der pathologischen Komplettremissionen nach neoadjuvanter Kombinationstherapie signifikant höher. In der Studie CheckMate 816, in der nur eine neoadjuvante Therapie verabreicht wurde, zeigte sich in diesen Fällen nach 5 Jahren eine Überlebenswahrscheinlichkeit von mehr als 90 %. Der Verzicht auf eine adjuvante Immuntherapie bei pathologischer Komplettremission wird daher aktuell intensiv diskutiert, insbesondere da es bislang keine Studie gibt, die explizit die Wirksamkeit eines neoadjuvanten oder perioperativen Konzeptes bei pathologischer Komplettremission miteinander verglichen hat. Damit geht zwangsläufig auch einher, dass der Bestimmung der therapieinduzierten Tumorregression nach neoadjuvanter Chemoimmuntherapie an NSCLC-Resektaten und dabei vor allem der Feststellung einer pathologischen Komplettremission im Alltag von Pathologinnen und Pathologen eine immer größere Bedeutung zukommt. Das Ausmaß der therapieinduzierten Tumorregression im Bereich des Primärtumors und der regionären Lymphknoten stellt dabei einen unabhängigen Prognosefaktor [9, 16, 25] und zugleich einen wichtigen primären bzw. sekundären Endpunkt in klinischen Studien [18] dar. Ziel dieses Artikels ist es, einen Überblick über die aktuellen Empfehlungen zur makroskopischen Aufarbeitung, die histologische Beurteilung und zum Staging von NSCLC-Resektaten nach neoadjuvanter Therapie zu geben.

## Makroskopische Aufarbeitung

**Abb. 1:**
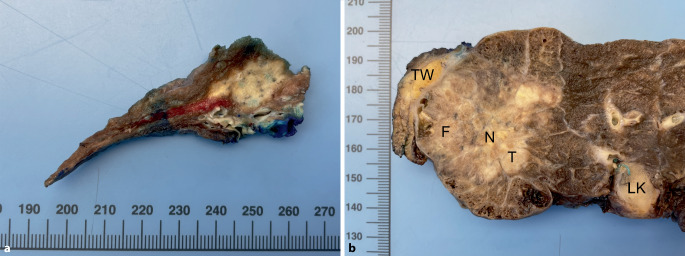
Pulmonales Adenokarzinom nach neoadjuvanter Therapie. Während sich in **a** lediglich ein unscharf begrenztes Narbenareal zeigt (histologisch „major pathologic response“), lassen sich in **b** bereits makroskopisch vitale Tumorresiduen abgrenzen. *F* Fibrose, *LK* intrapulmonale Lymphknotenmetastase, *N* Nekrose, *T* vitaler Tumor, *TW* Thoraxwand

Die makroskopische Aufarbeitung ist der erste wesentliche Schritt in der Befundung von NSCLC-Resektaten nach neoadjuvanter Therapie. Sofern für Pathologinnen und Pathologen Zugriff auf aktuelle Bildgebung besteht, kann eine Durchsicht der bildgebenden Befunde im Vorfeld des eigentlichen Präparatezuschnittes sehr hilfreich sein, um das tatsächliche Tumorbett zu identifizieren [6]. Insbesondere im Falle einer pathologischen Komplettremission („pathologic complete response“, pCR) ist makroskopisch unter Umständen nur noch ein diskretes Narbenareal sichtbar (Abb. 1a), das in manchen Fällen schwer zu identifizieren ist. In Fällen mit geringem Ansprechen auf die neoadjuvante Therapie ist das makroskopische Erscheinungsbild dem von unbehandelten Tumoren dagegen sehr ähnlich (Abb. 1b). Nach Identifikation sollte das Tumorbett zunächst in 3 Dimensionen gemessen werden.

Weissferdt et al. haben in einer aufwendigen und bisher einzigartigen Studie gezeigt, dass mit konventionellen Einbettschemata keine adäquate Bestimmung des Regressionsgrades möglich ist [29]. Um eine Genauigkeit von zumindest 90 % zu erreichen, ist entweder die Einbettung des gesamten Tumors oder (bei Tumoren ab 3 cm) von mindestens 21 Blöcken notwendig. Eine unabhängige Validierung dieser Parameter wurde bisher noch nicht publiziert. Basierend auf einem Expertenkonsensus, hat die IASLC (International Association for the Study of Lung Cancer) 2020 folgendes standardisiertes Vorgehen vorgeschlagen [27]: Ein Tumorbett von 3 cm oder weniger in der größten Ausdehnung sollte vollständig eingebettet werden, bei einem Tumorbett von größer als 3 cm zunächst der größte Querschnitt des Tumorbettes mit einer Dicke von etwa 0,5 cm sowie jeweils zusätzlich ein Block/cm Tumorbett. Falls die histologische Untersuchung keinen vitalen Resttumor zeigt, sollte auch das restliche Tumorbett aufgearbeitet werden. Die Schnitte in der Tumorperipherie sollten bei oben beschriebenem Vorgehen jeweils die Tumorgrenze mit zumindest 1 cm des angrenzenden nichtneoplastischen Lungenparenchyms erfassen, um die Ausdehnung des Tumorbettes ggf. histologisch verifizieren/korrigieren zu können (Tab. 1).

**Tab. 1:** Makroskopische und histologische Aufarbeitung von Resektaten nichtkleinzelliger Lungenkarzinome nach neoadjuvanter Therapie

Primärtumor		Lymphknoten	
Identifikation von Tumor/Tumorbett (ggf. Korrelation mit Bildgebung)		Lymphknoten (LK) identifizieren und nach anatomischen Leveln getrennt einbetten	
Messung in 3 Dimensionen		Nichtvorhandensein von LK im pathologischen Befundbericht angeben	
Schnittfläche mit dem größten Tumordurchmesser fotografieren		Eindeutig befallene LK:	mit max. Durchmesser von ≤ 2 cm: histologische Evaluation des *gesamten* LK
Tumordurchmesser ≤ 3 cm:	Histologische Evaluation des *gesamten* Tumors		mit max. Durchmesser von > 2 cm: Histologische Evaluation *einer zentralen Lamelle, *wenn histologisch kein vitaler Resttumor: Einbettung weiteren Gewebes, ggf. kompletter LK
Tumordurchmesser > 3 cm:	Histologische Evaluation einer *repräsentativen Scheibe der größten Tumorausdehnung *(Dicke 0,5 cm), ggf. Korrelation mit Fotodokumentation	Bei allen LK mindestens 2 Anschnitte/Objektträger	
	Wenn histologisch kein vitaler Resttumor: Einbettung weiteren Gewebes, ggf. komplettes Tumorbett		
Histologische Untersuchung von Tumorrandbereich und 1 cm des umgebenden Lungenparenchyms			
Multiple Tumoren:	intrapulmonale Metastasen: *ein* Regressionsgrad		
	unabhängige Primarien: getrennte Auswertung		

Zur makroskopischen Aufarbeitung von Lymphknoten bei NSCLC-Patienten nach neoadjuvanter Therapie liegen bislang keine detaillierten Studien vor. Der Expertenkonsens der IASLC empfiehlt die vollständige Einbettung von Lymphknoten bis zu einer Größe von 2 cm. Lymphknoten mit einer sehr großen Metastase bzw. einem Tumorbett > 2 cm können halbiert und eine zentrale Scheibe eingebettet werden [27], wobei in Analogie zum Primärtumor auch hier die Einbettung weiteren Gewebes notwendig ist für den Fall, dass sich auf den initialen Schnitten kein vitaler Resttumor mehr findet ([2]; Tab. 1).

## Histologische Regressionsgradbestimmung

**Abb. 2:**
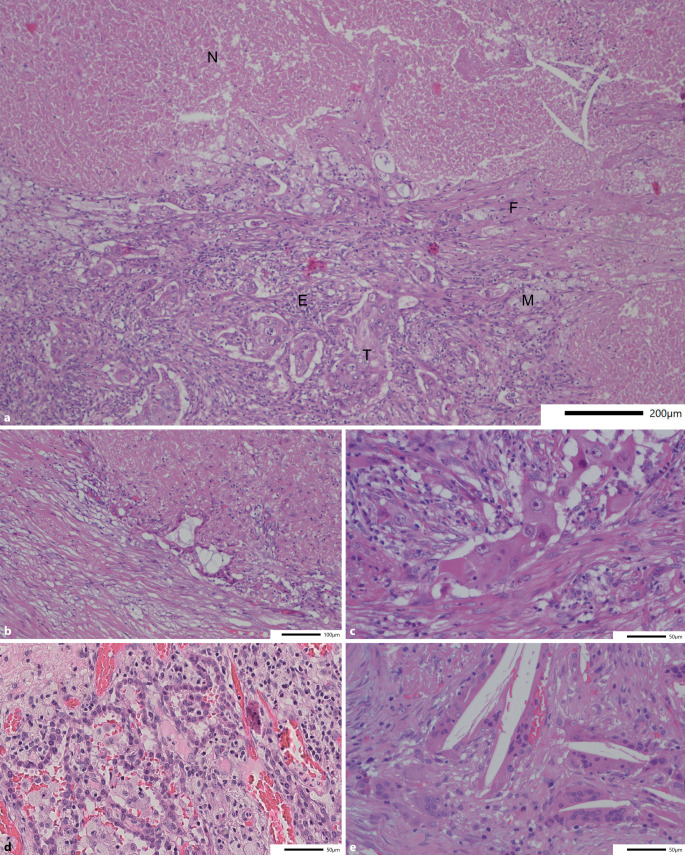
**a** Das regressiv veränderte pulmonale Adenokarzinom aus Abb. 1b (Vergr. 100:1, HE). *E* Stroma mit lymphozytärer Entzündung, *F* Fibrose, *M* Makrophagen, *N* Nekrose, *T* vitaler Tumor. **b** Derselbe Tumor aus **a **mit hier deutlich stärkeren regressiven Veränderungen und variabel kollagenfaserreichem, teils auch myxoidem Stroma (Vergr. 200:1, HE). **c** Adenokarzinom mit zytopathischen Veränderungen mit deutlicher Schwellung von Zellkernen und Zytoplasma (Vergr. 400:1, HE). **d** Reaktive Epithelatypien (Vergr. 400:1, HE). **e** Cholesterolspaltlücken mit resorptivzelliger Entzündung und mehrkernigen Riesenzellen (Vergr. 400:1, HE)

Das Tumorbett nichtkleinzelliger Lungenkarzinome nach neoadjuvanter Therapie setzt sich generell aus den 3 folgenden Komponenten zusammen: vitaler Resttumor („residual viable Tumor“, RVT), Stroma und Nekrose (Abb. 2a, b). Unter Bezug auf Pataer et al. [21] empfiehlt der Expertenkonsens der IASLC diese 3 Komponenten in 10 %-Schritten zu quantifizieren, wobei die Gesamtsumme 100 % ergeben sollte. Eine noch genauere Quantifizierung sollte nur vorgenommen werden, wenn die jeweilige Komponente weniger als 5 % des Tumorbettes ausmacht.

Auch wenn eine Abgrenzung der verschiedenen Komponenten des Tumorbetts in den meisten Fällen kaum Schwierigkeiten bereitet, gilt es, einige spezielle Aspekte zu berücksichtigen. Die nach neoadjuvanter Therapie verbliebenen vitalen Tumorzellen weisen häufig zytopathische Veränderungen in Form von Zell- und Kernvergrößerung auf (Abb. 2c; [15]), was die Abgrenzung von angrenzendem, reaktiv verändertem, nichtneoplastischem Lungenparenchym schwierig machen kann. Die atypische Hyperplasie der Typ-II-Pneumozyten (Abb. 2d) zeigt im Gegensatz zu Tumorzellen allerdings eine erhaltene Kern-Plasma-Relation. Zudem findet sich i. d. R. ein gradueller Übergang der Veränderungen zum angrenzenden Lungenparenchym und kein abrupter Sprung [23]. Der Vergleich mit dem angrenzenden nichtneoplastischen Lungenparenchym kann hier sehr hilfreich sein. Basierend auf dem für gewöhnlich praktisch fehlenden Ansprechen muzinöser Adenokarzinome auf neoadjuvante Therapie kann azelluläres Muzin als vitaler Tumor gewertet werden, sofern sich zumindest fokal darin an einer Stelle Tumorzellen nachweisen lassen [7, 27]. Liegt ausschließlich azelluläres Muzin ohne jeglichen Nachweis von Tumorzellen vor, sollte dies allerdings als regressives Stroma gewertet werden.

Das Stroma des Tumorbettes umfasst Fibrose und Entzündung – 2 Komponenten, die oft miteinander vermischt sind und daher zusammengefasst werden sollten [27]. Die Entzündung kann dabei sowohl aus chronischer Entzündung mit Lymphozyten, Plasmazellen oder lymphoiden Aggregaten als auch neutrophilen Granulozyten oder resorptivzelliger Entzündung mit Makrophagen oder xanthogranulomatöser Entzündung bestehen. Auch die Beschaffenheit des Stromas variiert. Es kann variabel kollagenfaserreich, fibroelastotisch, fibromyxoid oder sklerotisch sein.

## Mitteilung der stattgefundenen Behandlung durch die Klinik ist unabdingbar

Identische morphologische Veränderungen können auch therapieunabhängig im Rahmen einer spontanen Tumorregression beobachtet werden [17], sodass eine korrekte Einordnung nur unter ausreichender Kenntnis der Behandlungsvorgeschichte möglich und eine entsprechende Mitteilung durch die Klinik unabdingbar ist. Ein Auftreten therapieassoziierter Veränderungen, insbesondere in der Tumorperipherie, als möglicher Hinweis wurde zwar diskutiert [16], stellt aber kein verlässliches Kriterium dar [3, 7].

Es wurde berichtet, dass spontane Tumornekrosen im Gegensatz zu therapieinduzierten Nekrosen (bzw. Apoptosezonen) eine überwiegend im Randbereich gelegene granulozytäre Reaktion aufweisen und granulomartig angeordnete Cholesterinkristalllücken mit riesenzelliger Abräumreaktion im Rahmen der spontanen Tumorregression nur selten zur Darstellung kommen (Abb. 2e; [15]). Jedoch ist diese Unterscheidung schwierig und nicht validiert. Daher wird eine Unterscheidung verschiedener Nekrosetypen (therapieinduziert versus spontane Tumornekrose) im Alltag nicht empfohlen und jegliche Nekrose innerhalb des Tumorbettes als therapieassoziiert gewertet [27]. Für die korrekte Quantifizierung der Nekrose ist eine Korrelation mit der Makroskopie besonders wichtig. Insbesondere bei zentral zystisch-nekrotischen Tumoren besteht die Gefahr, dass in erster Linie die soliden Anteile in der Peripherie eingebettet werden und der vitale Tumoranteil damit überschätzt wird [27].

**Abb. 3:**
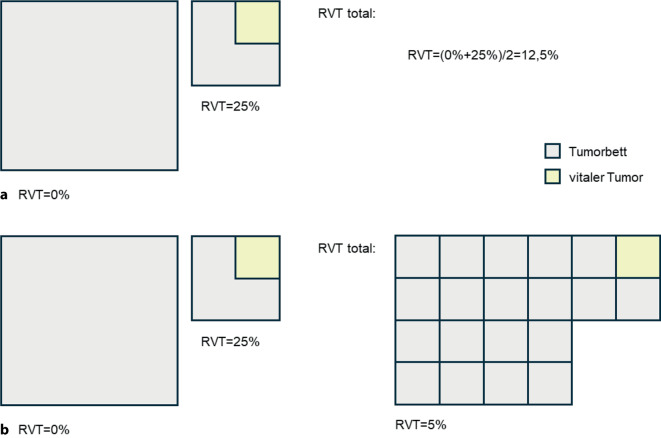
**a** Pro Schnitt wird der prozentuale Anteil des vitalen Resttumors (*RVT*) bestimmt, summiert und durch die Anzahl der Schnitte dividiert. **b** Beim gewichteten Ansatz wird zusätzlich auch bei jedem Schnitt der unterschiedliche Anteil an Tumorbett berücksichtigt (Abbildung modifiziert nach Saqi et al. [24])

Während die ersten Studien einen semiquantitativen Ansatz, d. h. ein ungefähres Abschätzen der Tumorbettkomponenten nach Augenmaß (sog. „eyeballing“), verwendet haben [16], haben neuere Studien den Gesamtprozentsatz der jeweiligen Komponenten durch Berechnung von Durchschnittswerten aus allen Tumorbettschnitten bestimmt (gewichteter Ansatz) (Abb. 3a; [21, 30]). Überdies hat sich jüngst auch ein gewichteter Ansatz etabliert, der zusätzlich die unterschiedliche Größe des Tumorbettes pro Schnitt berücksichtigt (Abb. 3b; [24]). Die beiden zuletzt genannten Ansätze zeigten in einer Interobserverstudie vergleichbare Ergebnisse, sodass die Anwendung des zeitaufwendigeren gewichteten Ansatzes im Alltag von den Experten der IASLC nur bei grenzwertigen Fällen empfohlen wird [7]. KI-basierte Verfahren kommen bislang nur vereinzelt in Studien zum Einsatz, haben aber ein großes Potential, die sehr arbeitsaufwendige Tumorregressionsbestimmung zukünftig zu erleichtern. Sie deuten darauf hin, dass menschliche Auswertende eher dazu tendieren, den vitalen Tumor zu überschätzen, da eine Trennung der Tumorzellen vom dazwischenliegenden Stroma für das menschliche Auge schwierig ist [8].

Für die Regressionsgraduierung beim nichtkleinzelligen Lungenkarzinom existieren 3 verschiedene Schemata: das von der aktuellen deutschen S3-Leitlinie empfohlene Junker-System [1], das IASLC-System [27] und das irPRC-System („immune-related pathologic response criteria“) [5]. Der prognostische Schwellenwert von 10 % vitalem Tumor geht dabei auf Junker et al. zurück [14, 16]. Keine oder nur spontane Tumorregression im Bereich von Primärtumor und mediastinalen Lymphknoten wird im Junker-System als Grad I definiert. Eine unvollständige therapieinduzierte Tumorregression mit mindestens 10 % vitalem Resttumor im Bereich des Primärtumors und/oder mehr als kleinherdiger Befall mediastinaler Lymphknoten entspricht einem Grad IIa, weniger als 10 % vitaler Resttumor im Bereich des Primärtumors und/oder kleinherdiger Befall mediastinaler Lymphknoten einem Grad IIb. Grad III ist definiert als vollständige therapieinduzierte Tumorregression ohne Nachweis vitalen Tumorgewebes im Bereich von Primärtumor und mediastinalen Lymphknoten. Im Scoringsystem der IASLC wird 10 % oder weniger vitaler Tumor im Bereich des Primarius als „major pathologic response“ (MPR) definiert. Das Fehlen vitaler Tumorzellen im Primarius und den Lymphknoten entspricht einem „pathologic complete response“ (pCR). Der Hauptunterschied zwischen dem vor allem im deutschsprachigen Raum verbreiteten Junker-System und dem IASLC-Scoring liegt dabei bei einem vitalen Tumor von genau 10 % (Tab. 2). Während die beiden zuletzt genannten Scoringsysteme explizit unabhängig vom neoadjuvanten Therapieschema (konventionell oder immunonkologisch) spezifisch beim nichtkleinzelligen Lungenkarzinom zur Anwendung kommen, lässt sich das irPRC-System auf alle Tumorarten anwenden, jedoch nur nach Vortherapie mit Immuncheckpoint-Inhibitoren [5]. Das Standardschema für NSCLC ohne Treibermutationen stellt jedoch die *Chemo*immunotherapie dar, weshalb irPRC eher nicht Anwendung finden sollte. Das Regressionsbett ist im Gegensatz zu den beiden anderen Scoringsystemen definiert als proliferative Fibrose mit Neovaskularisation und Zeichen der Immunaktivierung und des Zelltodes [10].

**Tab. 2:** Die Graduierungssysteme nach Junker und IASLC.

Grading-System			
Junker		IASLC	
I	Keine oder ausschließlich spontane Tumorregression im Bereich des Primärtumors und der regionären Lymphknoten	No MPR/pCR	> 10 % vitale Tumorzellen im Bereich des Primärtumors
IIa	Therapieinduzierte Tumorregression mit mindestens 10 % vitalem Resttumor im Bereich des Primärtumors und/oder mehr als kleinherdiger Nachweis vitalen Tumorgewebes in den regionären Lymphknoten		
IIb	Therapieinduzierte Tumorregression mit weniger als 10 % vitalem Resttumor im Bereich des Primärtumors und/oder mehr als kleinherdiger Nachweis vitalen Tumorgewebes in den regionären Lymphknoten	MPR	≤ 10 % vitale Tumorzellen im Bereich des Primärtumors
		MPR(N+)	Keine oder ≤ 10 % vitale Tumorzellen im Bereich des Primärtumors, aber vitale Tumorzellen in den regionären Lymphknoten
III	Komplette therapieinduzierte Tumorregression ohne Nachweis vitalen Tumorgewebes im Bereich des Primärtumors und der regionären Lymphknoten	pCR	Keine vitalen Tumorzellen im Bereich des Primärtumors und der regionären Lymphknoten

*MPR* „major pathologic response“, *pCR* „pathologic complete response“

Während das Junker-System ursprünglich keinen separaten Regressionsgrad für die Lymphknoten vergibt, sondern Fälle mit Nachweis von vitalem Resttumor innerhalb des Lymphknotens in die Gruppen Grad IIb und III mit eingruppiert, sieht das IASLC-System explizit einen zum Primärtumor analogen Graduierungsansatz mit Bestimmung des Prozentsatzes von vitalem Tumor, Nekrose und Stroma vor [27]. In Ergänzung zu den ursprünglichen IASLC-Empfehlungen wird empfohlen, Fälle mit 10 % vitalem Tumor im Bereich des Primarius oder pathologischer Komplettremission des Primarius (ypT0) und gleichzeitig jeweils Nachweis von vitalem Tumor in den Lymphknoten (ypN1, 2 oder 3) als „major pathologic response N+“ MPR(N+) zu klassifizieren [2].

**Abb. 4:**
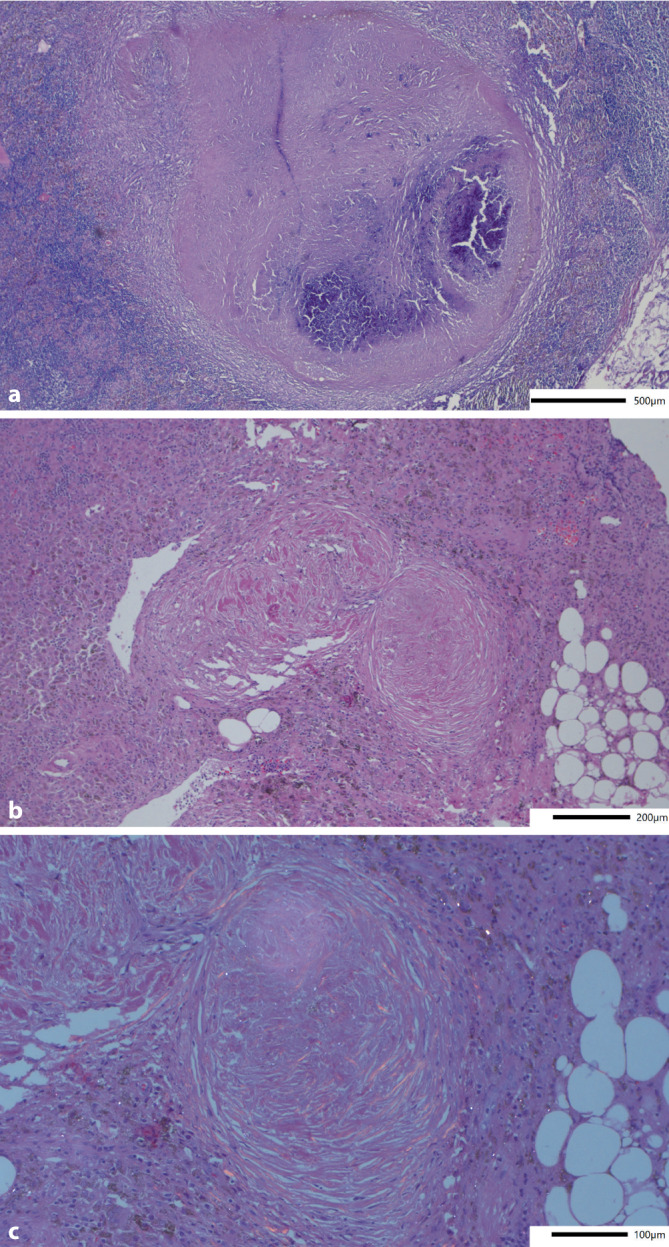
**a** Umschriebenes Narbenareal und Nekrose bei pathologischer Komplettremission eines pulmonalen Plattenepithelkarzinoms nach Chemoimmuntherapie (Vergr. 40:1, HE). **b** Hyalinschwieliges Knötchen bei Silikoanthrakose (Vergr. 100:1, HE) mit Nachweis doppeltbrechender feinkristalliner Präzipitate (**c** Vergr. 200:1, HE Polarisation)

Für die Diagnose einer pathologischen Komplettremission im Lymphknoten ist das Vorhandensein einer umschriebenen Narbe oder Tumornekrose in Abwesenheit vitaler Tumorzellen erforderlich (Abb. 4a). (Siliko‑)Anthrakoseknoten und Granulome sind differentialdiagnostisch davon abzugrenzen (Abb. 4b). Das Vorhandensein von Anthrakosepigment oder der Nachweis doppeltbrechender Kristalle können dabei hilfreich sein (Abb. 4c) ebenso wie die Kenntnis der Lymphknotenstationen mit prätherapeutischem Karzinomnachweis [27]. Der Nachweis von ausschließlich azellulärem Muzin innerhalb des Lymphknotens (ohne vitale Tumorzellen) sollte als pathologische Komplettremission gewertet werden. Entzündung ist innerhalb des lymphatischen Parenchyms naturgemäß schwer zu beurteilen und bisher fehlen Studien zur Durchführbarkeit einer eindeutigen Tumorbettbestimmung.

Zwei Studien konnten zeigen, dass ein MPR im Lymphknoten einen unabhängigen prognostischen Faktor darstellt [19, 22]. Was den vitalen Tumor anbelangt, schlagen beide Studien mit 8 % bzw. 70 % allerdings sehr unterschiedliche Cutoff-Werte für einen Lymphknoten-spezifischen MPR vor. Angesichts dieser deutlichen Diskrepanz und der relativ kleinen Fallzahlen in beiden Arbeiten (*n* = 336 bzw. *n* = 75) sind hier noch weitere Studien dringend notwendig.

## Staging

Das Messen der Tumorgröße und die Bestimmung des ypT-Stadiums kann an NSCLC-Resektaten nach neoadjuvanter Therapie herausfordernd sein. Ist ein einzelner diskreter Fokus aus vitalem Tumor vorhanden, lässt sich dieser problemlos messen. Häufig liegt aber aufgrund eines heterogenen Therapieansprechens nicht ein einzelner Fokus vor, sondern multiple Inseln aus vitalem Tumor, gemischt mit Stroma und Nekrosearealen. In diesem Fall empfiehlt die IASLC, die Tumorgröße und das ypT-Stadium durch Multiplikation des Prozentsatzes aus vitalem Tumor (RVT) mit dem maximalen Durchmesser des Tumorbettes zu bestimmen [26].

**Abb. 5:**
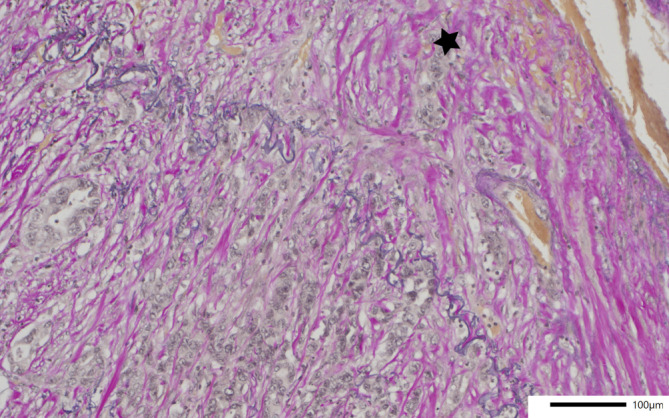
Infiltration der Pleura visceralis: Voraussetzung ist, dass vitale Tumorzellen tatsächlich die Lamina elastica externa durchbrechen (PL1; Vergr. 200:1, EVG; *Stern*: Tumorzellen jenseits der Elastica externa)

Was die Infiltration TNM-relevanter anatomischer Strukturen (Pleura, Brustwand u. a.) anbelangt, sollte nur der Nachweis vitaler Tumorzellen innerhalb dieser Strukturen als Infiltration gewertet werden (Abb. 5). Eine alleinige Ausdehnung des regressiven Tumorbettes, d. h. von Stroma/Entzündung und/oder Nekrose auf eine der relevanten anatomischen Strukturen reichen dazu nicht aus.

Findet sich zusätzlich zum invasiven Karzinom eine In-situ-Komponente, gilt es, wie bei nicht vorbehandelten Patienten beide Komponenten im Befund zu berichten, wobei für das ypT-Stadium allein die invasive Komponente ausschlaggebend ist. Im Fall von multiplen, klonal unabhängigen Tumoren sollte für jeden Tumor getrennt die Regression angegeben werden [27].

Für die Bestimmung des ypN-Stadiums (ypN0-3) und die Angabe des R‑Status sollten die geltenden TNM-Regeln angewendet werden [2].

## Aktuelle Praxis im deutschsprachigen Raum

Eine multizentrische Studie hat kürzlich die Praxis des Regressionsgradings an NSCLC-Resektaten in Deutschland untersucht [11]. Untersucht wurden dabei 122 Fälle aus 8 großen thoraxchirurgischen Zentren. Hierbei konnten auch in der Real-world-Situation gute Ansprechraten der Chemoimmuntherapie mit einer pathologischen Komplettremission (pCR nach IASLC/RGIII nach Junker) in 45,9 % der Fälle gezeigt werden. Allerdings war nur eine schwache Korrelation von PD-L1 TPS („tumor proportion score“) am prätherapeutischen Biopsat und dem Therapieansprechen (gemessen als Prozentsatz des vitalen Resttumors, RVT) am Resektat nachweisbar. In Fällen mit pathologischer Komplettremission fand sich eine breite Streuung der prätherapeutischen TPS von 0 bis 100 % (median, 60 %) und eine pathologische Komplettremission wurde in 40 % der Fälle mit einem prätherapeutischen TPS von < 1 % beobachtet. Neue prädiktive Biomarker, die ein Ansprechen von NSCLC-Patienten auf Chemoimmuntherapie mit größerer Zuverlässigkeit vorhersagen, sind daher dringend notwendig.

Sowohl hinsichtlich der Makroskopie als auch bei der histologischen Befundung zeigte sich ein in der Praxis uneinheitliches Vorgehen: In 18 % der Fälle waren Tumoren mit einem max. Durchmesser ≤ 3 cm nicht vollständig eingebettet worden. Sowohl das System nach Junker als auch das IASLC-System sind im deutschsprachigen Raum sehr verbreitet (97,7 %) mit leichtem Überwiegen des Junker-Gradings, wobei beide Systeme weitgehend übereinstimmen. Eine Diskrepanz fand sich nur in einem der 133 Fälle mit exakt 10 % RVT. Was die Regressionsgraduierung der Lymphknoten anbelangt, war das Vorgehen weniger einheitlich. Eine separate Regression für die Lymphknoten wurde lediglich in 60,2 % der Fälle angegeben, wobei eine separate Graduierung der Lymphknoten gerade bei Fällen mit unterschiedlichem Ansprechen von Primarius und Lymphknoten hilfreich sein könnte, um in Zukunft die prognostische Bedeutung eines unterschiedlichen Ansprechens dieser beiden Kompartimente besser einschätzen zu können.

## Zusammenfassung

Wesentlich für die Befundung von NSCLC-Resektaten nach neoadjuvanter Therapie ist eine sorgfältige makroskopische Aufarbeitung mit adäquater Einbettung des Tumors (Tumoren ≤ 3 cm vollständig, > 3 cm größter Querschnitt und weitere Entnahmen pro cm). Histologisch besteht das Tumorbett aus dem vitalen Tumor, Stroma/Entzündung und Nekrose. Diese Komponenten sollten in 10 %-Schritten quantifiziert und der RVT als kontinuierliche Variable angegeben werden (1 %-Schritte nur bei < 5 % RVT). Zusätzlich sollte ein Regressionsgrad nach IASLC (pCR/MPR) bzw. nach Junker vergeben werden.

Die aktuellen Empfehlungen beruhen teilweise auf einem Expertenkonsensus, sodass noch weitere Daten gesammelt werden müssen, die ggf. in Zukunft zu einer Anpassung der Empfehlungen führen. Eine breite Anwendung der IASLC-Empfehlungen ist wünschenswert, um eine Standardisierung und weltweite Übertragbarkeit der pathologischen Befunde zu gewährleisten.

KI-basierte Verfahren könnten in Zukunft helfen, den Regressionsgrad noch genauer zu bestimmen und eine präzisere prognostische Stratifizierung von Patienten nach neoadjuvanter Therapie ermöglichen. Grundvoraussetzung für die Anwendung KI-basierter Verfahren ist allerdings eine durchgängige Digitalisierung der Schnittpräparate, was aktuell lediglich in wenigen pathologischen Instituten routinemäßig der Fall ist – wenn auch mit steigender Tendenz. Die digitalisierten Schnittpräparate der Multicenterstudie Re-GraDE könnten darüber hinaus den Grundstein für eine virtuelle Sammlung von OP-Präparaten nach neoadjuvanter Chemoimmuntherapie darstellen. Weitere Biomarker wie die Messung der ctDNA (zirkulierende Tumor-DNA) im Blut könnten überdies künftig eine wertvolle Ergänzung sein.

Empfehlungen zur Regressionsgradbestimmung an (extrapulmonalen) Fernmetastasen existieren aktuell nicht, da eine kurativ intendierte neoadjuvante Therapie aktuell nur für Patienten bis zum UICC-Stadium IIIA empfohlen ist und Fernmetastasen ausschließt. Durch neue Therapiekonzepte, insbesondere für oligometastasierte Patienten, könnte dies allerdings künftig ebenfalls von Bedeutung sein.

## Fazit für die Praxis

- Die neoadjuvante Chemoimmuntherapie bei operablem nichtkleinzelligem Lungenkarzinom ist bereits leitlinienkonformer Standard in der Thoraxonkologie.
- Die adäquate makroskopische Aufarbeitung ist sehr wichtig: Tumoren ≤ 3 cm sollten vollständig eingebettet werden, bei Tumoren > 3 cm der größte Querschnitt und weitere Entnahmen pro cm.
- Der prozentuale Anteil vitalen Tumors sollte in 10 %-Schritten angegeben und der Regressionsgrad nach IASLC (und evtl. Junker) berichtet werden.
- Eine Infiltration TNM-relevanter Strukturen sollte nur diagnostiziert werden, wenn sich dort tatsächlich vitaler Tumor nachweisen lässt.
- Zur Standardisierung der Befunde ist die breite Anwendung der IASLC-Empfehlungen wünschenswert
- Neue prädiktive Biomarker, die ein Ansprechen auf neoadjuvante Therapie vorhersagen, werden dringend benötigt.

## References

[1] Anonymous S3-Leitlinie Prävention, Diagnostik, Therapie und Nachsorge des Lungenkarzinoms. In:

[2] Berezowska S, Dacic S, Weissferdt A et al (2025) PT1.07.01 IASLC Multidisciplinary Recommendations for Pathological Response Evaluation of Resected Lymph Nodes After Neoadjuvant Therapy in NSCLC. Journal of Thoracic Oncology 20:S600

[3] Blaauwgeers JL, Kappers I, Klomp HM et al (2013) Complete pathological response is predictive for clinical outcome after tri-modality therapy for carcinomas of the superior pulmonary sulcus. Virchows Arch 462:547–556 23549732 10.1007/s00428-013-1404-6

[4] Cascone T, Spicer JD, Provencio Pulla M (2024) Perioperative Nivolumab in Resectable Lung Cancer Reply. N Engl J Med 391:573–574 39115074 10.1056/NEJMc2407267

[5] Cottrell TR, Thompson ED, Forde PM et al (2018) Pathologic features of response to neoadjuvant anti-PD‑1 in resected non-small-cell lung carcinoma: a proposal for quantitative immune-related pathologic response criteria (irPRC). Ann Oncol 29:1853–1860 29982279 10.1093/annonc/mdy218 PMC6096736

[6] Dacic S (2025) Neoadjuvant Therapy and Lung Cancer: Role of Pathologists. Arch Pathol Lab Med 149:e78–e81 39448058 10.5858/arpa.2024-0203-RA

[7] Dacic S, Travis W, Redman M et al (2023) International Association for the Study of Lung Cancer Study of Reproducibility in Assessment of Pathologic Response in Resected Lung Cancers After Neoadjuvant Therapy. J Thorac Oncol 18:1290–1302 37702631 10.1016/j.jtho.2023.07.017

[8] Dacic S, Travis WD, Giltnane JM et al (2024) Artificial Intelligence-Powered Assessment of Pathologic Response to Neoadjuvant Atezolizumab in Patients With NSCLC: Results From the LCMC3 Study. J Thorac Oncol 19:719–731 38070597 10.1016/j.jtho.2023.12.010

[9] Deutsch JS, Cimino-Mathews A, Thompson E et al (2024) Association between pathologic response and survival after neoadjuvant therapy in lung cancer. Nat Med 30:218–228 37903504 10.1038/s41591-023-02660-6 PMC10803255

[10] Deutsch JS, Scolyer RA, Burton E et al. Updated pan-tumor guidelines for neoadjuvant scoring of pathologic response: A joint SITC and INMC effort. Annals of Oncology 10.1016/j.annonc.2025.10.018 PMC13318440 41469296

[11] Elsner F, Kumpers C, Ott G et al (2025) Current practice of pathologic response assessment following chemoimmunotherapy for non-small cell lung cancer (NSCLC) in Germany: first real-world data from the multicentre Re-GraDE study. Histopathology 87:869–879 40911027 10.1111/his.15550 PMC12605775

[12] Forde PM, Spicer JD, Provencio M et al (2025) Overall Survival with Neoadjuvant Nivolumab plus Chemotherapy in Lung Cancer. N Engl J Med 393:741–752 40454642 10.1056/NEJMoa2502931

[13] Heymach JV, Harpole D, Mitsudomi T et al (2023) Perioperative Durvalumab for Resectable Non-Small-Cell Lung Cancer. N Engl J Med 389:1672–1684 37870974 10.1056/NEJMoa2304875

[14] Junker K, Langner K, Klinke F et al (2001) Grading of tumor regression in non-small cell lung cancer : morphology and prognosis. Chest 120:1584–1591 11713138 10.1378/chest.120.5.1584

[15] Junker K, Muller KM, Abker S et al (2004) Cellular changes in non-small cell lung cancer after neoadjuvant therapy. Pathologe 25:193–201 15138700 10.1007/s00292-003-0643-8

[16] Junker K, Thomas M, Schulmann K et al (1997) Tumour regression in non-small-cell lung cancer following neoadjuvant therapy. Histological assessment. J Cancer Res Clin Oncol 123:469–477 9341895 10.1007/BF01192200 PMC12201412

[17] Kerr KM, Johnson SK, King G et al (1998) Partial regression in primary carcinoma of the lung: does it occur? Histopathology 33:55–63 9726050

[18] Lee JM, Kim AW, Marjanski T et al (2021) Important Surgical and Clinical End Points in Neoadjuvant Immunotherapy Trials in Resectable NSCLC. JTO Clin Res Rep 2:100221 34746882 10.1016/j.jtocrr.2021.100221 PMC8552106

[19] Liu X, Sun W, Wu J et al (2021) Major pathologic response assessment and clinical significance of metastatic lymph nodes after neoadjuvant therapy for non-small cell lung cancer. Mod Pathol 34:1990–1998 34253867 10.1038/s41379-021-00871-1

[20] Lu S, Zhang W, Wu L et al (2024) Perioperative Toripalimab Plus Chemotherapy for Patients With Resectable Non-Small Cell Lung Cancer: The Neotorch Randomized Clinical Trial. JAMA 331:201–211 38227033 10.1001/jama.2023.24735 PMC10792477

[21] Pataer A, Kalhor N, Correa AM et al (2012) Histopathologic response criteria predict survival of patients with resected lung cancer after neoadjuvant chemotherapy. J Thorac Oncol 7:825–832 22481232 10.1097/JTO.0b013e318247504a PMC3465940

[22] Pataer A, Weissferdt A, Vaporciyan AA et al (2021) Evaluation of Pathologic Response in Lymph Nodes of Patients With Lung Cancer Receiving Neoadjuvant Chemotherapy. J Thorac Oncol 16:1289–1297 33857666 10.1016/j.jtho.2021.03.029 PMC11996033

[23] Rekhtman N (2020) “Napoleon Hat” Sign: A Distinctive Cytologic Clue to Reactive Pneumocytes. Arch Pathol Lab Med 144:443–445 31971464 10.5858/arpa.2019-0615-SA

[24] Saqi A, Leslie KO, Moreira AL et al (2022) Assessing Pathologic Response in Resected Lung Cancers: Current Standards, Proposal for a Novel Pathologic Response Calculator Tool, and Challenges in Practice. JTO Clin Res Rep 3:100310 35498382 10.1016/j.jtocrr.2022.100310 PMC9044000

[25] Thomas M, Rube C, Semik M et al (1999) Impact of preoperative bimodality induction including twice-daily radiation on tumor regression and survival in stage III non-small-cell lung cancer. J Clin Oncol 17:1185 10561177 10.1200/JCO.1999.17.4.1185

[26] Travis WD, Asamura H, Bankier AA et al (2016) The IASLC Lung Cancer Staging Project: Proposals for Coding T Categories for Subsolid Nodules and Assessment of Tumor Size in Part-Solid Tumors in the Forthcoming Eighth Edition of the TNM Classification of Lung Cancer. J Thorac Oncol 11:1204–1223 27107787 10.1016/j.jtho.2016.03.025

[27] Travis WD, Dacic S, Wistuba I et al (2020) IASLC Multidisciplinary Recommendations for Pathologic Assessment of Lung Cancer Resection Specimens After Neoadjuvant Therapy. J Thorac Oncol 15:709–740 32004713 10.1016/j.jtho.2020.01.005 PMC8173999

[28] Wakelee H, Liberman M, Kato T et al (2023) Perioperative Pembrolizumab for Early-Stage Non-Small-Cell Lung Cancer. N Engl J Med 389:491–503 37272513 10.1056/NEJMoa2302983 PMC11074923

[29] Weissferdt A, Leung CH, Lin H et al (2024) Pathologic Processing of Lung Cancer Resection Specimens After Neoadjuvant Therapy. Mod Pathol 37:100353 37844869 10.1016/j.modpat.2023.100353 PMC10841500

[30] Weissferdt A, Pataer A, Vaporciyan AA et al (2020) Agreement on Major Pathological Response in NSCLC Patients Receiving Neoadjuvant Chemotherapy. Clin Lung Cancer 21:341–348 32279936 10.1016/j.cllc.2019.11.003 PMC7305995

[31] Welcker K, Jonigk D, Kropf-Sanchen C et al (2025) Neoadjuvant therapy for resectable non-small cell lung cancer. Pneumologie 79:16–24 39642922 10.1055/a-2465-4830 PMC11753866

[32] Yu H, Boyle TA, Zhou C et al (2016) PD-L1 Expression in Lung Cancer. J Thorac Oncol 11:964–975 27117833 10.1016/j.jtho.2016.04.014 PMC5353357

